# Low Sensitivity of Real Time PCRs Targeting Retrotransposon Sequences for the Detection of *Schistosoma japonicum* Complex DNA in Human Serum

**DOI:** 10.3390/pathogens10081067

**Published:** 2021-08-22

**Authors:** Hagen Frickmann, Ulrike Loderstädt, Beatrice Nickel, Sven Poppert, Peter Odermatt, Somphou Sayasone, Marjan Van Esbroeck, Isabel Micalessi, Lieselotte Cnops, Poom Adisakwattana, Gérard Leboulle, Olfert Landt, Thorsten Thye, Egbert Tannich

**Affiliations:** 1 Department of Microbiology and Hospital Hygiene, Bundeswehr Hospital Hamburg, 20359 Hamburg, Germany; 2 Department of Medical Microbiology, Virology and Hygiene, University Medicine Rostock, 18057 Rostock, Germany; 3 Department of Hospital Hygiene & Infectious Diseases, University Medicine Göttingen, 37075 Göttingen, Germany; ulrike.loderstaedt1@med.uni-goettingen.de; 4 Swiss Tropical and Public Health Institute, 4051 Basel, Switzerland; beatrice.nickel@swisstph.ch (B.N.); sven@poppert.eu (S.P.); peter.odermatt@swisstph.ch (P.O.); 5 University of Basel, 4001 Basel, Switzerland; 6 Lao Tropical and Public Health Institute, Vientiane Capital 01000, Laos; somphou.sayasone@yahoo.com; 7 Department of Clinical Sciences, Institute of Tropical Medicine, 2000 Antwerp, Belgium; mvesbroeck@itg.be (M.V.E.); imicalessi@itg.be (I.M.); lcnops@itg.be (L.C.); 8 Department of Helminthology, Faculty of Tropical Medicine, Mahidol University, Bangkok 10400, Thailand; poom.adi@mahidol.edu; 9 TIB MOLBIOL, 12103 Berlin, Germany; gleboulle@tib-molbiol.de (G.L.); olandt@tib-molbiol.de (O.L.); 10 Department Infectious Disease Epidemiology, Bernhard Nocht Institute for Tropical Medicine Hamburg, 20359 Hamburg, Germany; thye@bnitm.de; 11 National Reference Centre for Tropical Pathogens, Bernhard Nocht Institute for Tropical Medicine Hamburg, 20359 Hamburg, Germany

**Keywords:** *Schistosoma mekongi*, *Schistosoma malayensis*, hybridization probe, test evaluation, diagnosis, retrotransposon, multi-copy target, schistosomiasis, molecular diagnostics, test accuracy

## Abstract

While hybridization probe-based real-time PCR assays targeting highly repetitive multi-copy genome sequences for the diagnosis of *S. mansoni* complex or *S. haematobium* complex from human serum are well established, reports on the evaluation of respective assays for the identification of *S. japonicum* complex DNA in human serum are scarce. Here, we assessed the potential use of the retrotransposon sequences *SjR2* and *SjCHGCS19* from *S. japonicum*, *S. mekongi* and *S. malayensis* for the diagnosis of Asian *Schistosoma* infections. Based on available *S. japonicum* sequences and newly provided *S. mekongi* and *S. malayensis* sequences, hybridization probe-based real-time PCRs targeting *SjR2* and *SjCHGCS19* of the *S. japonicum* complex were designed both as consensus primer assays as well as multi-primer assays for the coverage of multiple variants of the target sequences. The assays were established using plasmids and *S. mekongi* DNA. While the consensus primer assays failed to detect *S. mekongi* DNA in human serum samples, the multi-primer assays showed positive or borderline positive results but only in 9.8% (6/61) of serum samples from patients with confirmed *S. mekongi* infections. Some cross-reactions with samples positive for *S. mansoni* or *S. haematobium* were observed but with the *SjCHGCS19*-PCR only. In spite of the low sensitivity, the presented experience may guide future evaluations of *S. japonicum-*complex-specific PCRs from human serum.

## 1. Introduction

Species of the *Schistosoma japonicum* complex with human pathogenic potential comprise *S. mekongi* and the closely related *S. malayensis* next to *S. japonicum sensu stricto* [1,2]. As a result of extensive ongoing *Schistosoma*-control and elimination programs in endemic settings [3,4], the quantitative disease burden due to *S. japonicum* complex is considerably lower compared to African schistosomiasis [5] caused by *S. haematobium* complex and *S. mansoni* complex [2], respectively, or compared to Latin American schistosomiasis caused by *S. mansoni* [5]. To eradicate residual parasite circulation, sensitive diagnostic tests are required [6].

For the African *Schistosoma* species, highly sensitive real-time PCRs targeting the multi-copy sequences *Sm1-7* for *S. mansoni* complex as well as *Dra1* for *S. haematobium* complex in human serum have been developed [2,7,8,9,10] and well evaluated using sera from travel returnees [11,12] and individuals in endemic areas [13,14]. These assays provide highly sensitive tools for screening and facilitate the identification of both species complexes in parallel in human serum samples as a one-tube multiplex approach [12,14]. Currently, its potential usefulness for schistosomiasis surveillance and control activities is controversially discussed [13,15,16]. In contrast, its application for the diagnosis of early infections prior to seroconversion and prior to the excretion of eggs, as well as for the discrimination of active infections from old serological responses to infections that have been successfully cured long ago, is considered as well established [12,13,14,15,16].

For *S. japonicum*, the retrotransposon *SjR2* has been described at the start of the millennium as a highly repetitive genetic element, which might be a suitable PCR target for sensitive molecular diagnostic approaches [17]. In detail, as many as 10,000 *SjR2* repeats per genome have been reported, representing about 14% of the *S. japonicum* genome [18]. In 2012, those estimations were corrected to a genome proportion of 4.43%, corresponding to about 400 complete copies and 23,755 partial copies [19]. In the same study [19], the retrotransposon *SjCHGCS19*, which represents a genome proportion of 4.09% with 793 complete copies and 17,373 partial copies, was identified as another potential multi-copy PCR target. However, *SjCHGCS19* is not specific for *S. japonicum*, as it is also present in *S. mansoni* [19].

Surprisingly, reports on the introduction, standardization and evaluation of diagnostic real-time PCR assays targeting repetitive genetic elements of *S. japonicum* complex in human serum are quite scarce. Focusing on the *SjCHGCS19* target, nested PCR but not real-time PCR was described to identify *S. japonicum* DNA in serum samples [19]. Traditional gel-based PCRs, nested PCRs as well as SYBR Green-based but not probe-based real-time PCRs targeting *SjR2* have been used to identify *S. japonicum* in tissue and animal sera [20,21,22,23,24,25,26]. Other molecular diagnostic approaches targeting *SjR2* sequence fragments of *S. japonicum* include loop-mediated isothermal amplification (LAMP) [27,28], recombinase polymerase amplification [29], and droplet digital PCR [30], respectively.

To our knowledge, there is a lack of well-evaluated modern hybridization probe-based real-time PCR assays for the diagnosis of *S. japonicum* infections by targeting the highly repetitive genetic elements *SjR2* or *SjCHGCS19*. Moreover, the described assays have not been adapted to cover *S. mekongi* and *S. malayensis*, two further *Schistosoma* spp. of the *S. japonicum* complex pathogenic to humans, because respective genomic information from these two species has not been available so far [31]. In this study, we aimed at establishing hybridization-probe real-time PCR assays targeting *SjR2* and *SjCHGCS19* for the diagnosis of infections with parasites of the *S japonicum* complex in human serum samples.

## 2. Materials and Methods

### 2.1. Samples

A total of 61 residual volumes (200 µL each) of serum samples from patients with microscopically proven *S. mekongi* infection were included as positive controls in the assessment. The residual serum samples had been stored frozen at −80 °C up to 10 years prior to the assessment and freeze-thawed several times. These serum samples had originally been collected for serological testing from the local population (n = 60) [32,33] in the course of studies conducted in the endemic area in Lao People’s Democratic Republic (PDR) as well as from a Belgian traveler returning from Lao PDR (n = 1) [34]. *S. mekongi* eggs were diagnosed, and the egg number per gram was counted by experienced microscopists in stool samples taken in parallel from all patients (mean: 51 eggs/gram, standard deviation: 144 eggs/gram, median: 18 eggs/gram, interquartile range IQR: (12, 30 eggs/gram), minimum-maximal (6, 1010 eggs/gram)). As detailed elsewhere [32,33,34], positive serological results had been observed in 40 out of 61 (65.6%) patients. Concomitant helminth infections were proven by the detection of *Opisthorchis viverrini* eggs (n = 32 stool samples), hookworm eggs (n = 23 stool samples) and *Trichuris trichiura* eggs (n = 1 stool sample), respectively.

Negative control samples comprised 50 residual serum samples from travel returnees with *S. mansoni* and/or *S. haematobium* infections as described in a previous study [12], which had also been stored at −80 °C for several years prior to the assessments. As detailed elsewhere [12], positive serum PCR for *S. haematobium* had been obtained in 21 instances (mean cycle threshold (Ct) value 33, standard deviation (SD) 5), positive serum PCR for *S. mansoni* in 33 instances (mean Ct 33, SD 4), with 8 mixed infections. Detection of anti-schistosomal antibodies had shown positive results in 27/50 instances (54%) next to another borderline positive result (2%) [12]. *S. mansoni* eggs had been observed in stool samples in two of those 50 patients, *S. haematobium* eggs in urine of three patients as well as in sperm of an additional patient, respectively, without quantification attempts.

To ensure sufficient DNA quality of the historical serum samples, real-time PCR targeting the human 18S rRNA gene, as described previously [35,36], had been sporadically performed with arbitrarily chosen study samples and fresh serum sample residuals from the diagnostic routine, showing no relevant Ct value shifts in the range of 24 ± 2. 

Further, two urine samples with positive real-time PCR for *S. haematobium* (Ct 25 and Ct 30, respectively) were included as additional negative controls. In the sample with Ct 25, *S. haematobium* eggs had been microscopically detected as well.

In line with ethical requirements for this test evaluation study, no patient-specific data can be presented, which is an admitted violation of the STARD (Standards for Reporting Diagnostic Accuracy) criteria [37].

### 2.2. Nucleic acid Extraction

Nucleic acids had been extracted from the complete 200 µL residual sample volumes using the QIAamp circulating nucleic acid kit (Qiagen, Hilden, Germany) according to the manufacturer’s instructions [8].

### 2.3. Real-Time PCRs

The hybridization-probe-based real-time PCR assays targeting the retrotransposons *SjR2* and *SjCHGCS19* were designed according to the suggestions from the original publication by Driscoll and colleagues [17] as well as by Guo and colleagues [19]. As available sequence information, however, was restricted to *S. japonicum sensu stricto* (e.g., GenBank accession numbers AF412214.1, AY027869.1, FN293022.1, FN293026.1, FN293027.1 for *SjR2* and FN356221.1 for *SjCHGCS19*, respectively), matching contigs from a short-read next generation sequencing (NGS) analysis of *S. mekongi* and *S. malayensis* cells taken from the reference material at the Bernhard Nocht Institute for Tropical Medicine Hamburg, which is the German National Reference Center for Tropical Pathogens, were also considered for the choice of the oligonucleotides. *SjR2*-like and *SjCHGCS19*-like sequences in *S. mekongi* and *S. malayensis* were identified with the software BLASTN 2.10.0+ as described recently [38]. The identified contigs are shown in Appendix A Table A1.

As the newly identified sequences were in part not optimally matched by the primer oligonucleotides suggested previously [17,19], new real-time PCR oligonucleotides were designed with the aim of optimally covering all three pathogenic species within the *S. japonicum* complex and also to match the considerable sequence variability within the retrotransposons *SjR2* and *SjCHGCS19*. For these purposes, both consensus PCRs and multi-primer PCRs to cover a broader range as sequence variants were designed in silico.

For initial test optimization, plasmids with a pEX-A128 backbone encircling the insert sequence were used. The *SjR2*-plasmid insert comprised the new sequence from the Appendix A Table A1, while the GenBank accession number FN356221.1 sequence was used for the *SjCHGCS19*-plasmid. After optimization, both the consensus and the multi-primer PCRs showed a limit of detection within the 10^3^ copies per µL eluate range for the *SjR2* target and within the 10^2^ copies per µL eluate range for the *SjCHGCS19* target when performed with the plasmids. Only after those pre-assessments, the protocols were compared with DNA from *S. mekongi* worms grown in the culture at the Mahidol University in Bangkok, Thailand (see results).

The master mix of all real-time PCRs comprised HotStar Taq Mastermix (Qiagen, Hilden, Germany) with a total Mg^2+^ concentration of 6.0 mM, 0.5 pmol/µL of each primer, 0.3 pmol/µL of the probe, 0.005 mg/mL bovine serum albumin, and 5.0 µL DNA eluate in total volumes of 20 µL, respectively. The run protocols were as follows: Heating to 95 °C for 15 minutes followed by 50 cycles of denaturation at 95 °C for 15 s, annealing for 60 s at 60 °C and amplification at 72 °C for 60 s each, followed by cooling to 40 °C for 20 s. The PCRs were run on magnetic induction cyclers (MIC; Bio Molecular Systems Ltd., London, UK). Inhibition of the PCR reactions was excluded with a protocol based on the amplification of Phocid herpes virus DNA as described previously [39]. All PCRs were run with a plasmid-based positive control and a PCR-grade water-based negative control.

Positive PCR results were defined by cycle threshold (Ct) values ≤35 and exponential amplification. In the case of Ct values >35 and/or atypical shapes of the amplification curves, the results were considered borderline positive.

### 2.4. Statistics

Due to the low number of available samples for the evaluation, the results were descriptively presented only.

### 2.5. Ethics

Ethical clearance for the anonymous use of residual sample materials for the test assessments was provided by the medical association of Hamburg, Germany (reference number: WF-011/19) on 11th March 2019 without a need for informed consent. The evaluation study was performed in line with the guidelines of the Declaration of Helsinki.

## 3. Results

### 3.1. Adaptation of the Oligonucleotide Compositions to Increase the Sensitivity of S. japonicum Complex PCR

Due to the lack of sequence information in public databases corresponding to *S. japonicum* retrotransposons *SjR2* [17] and *SjCHGCS19* [19] in *S. mekongi* and *S. malayenis*, the genomes of the latter two species were partially sequenced (see methods). This revealed additional sequence information to design new consensus primers and probes covering all three species of the *S. japonicum* complex. In addition, as there is considerable sequence variability of the different retrotransposon copies even within the same species, various primer combinations were introduced into multi-primer PCR assays in order to increase sensitivity when applied to DNA extracted from helminth material instead of plasmids which are usually used as positive controls.

Compared to consensus real-time PCR with helminth DNA of *S. mekongi*, sensitivity could be increased by 0.5 and 6.6 cycles for *SjR2* and *SjCHGCS19* sequences, respectively, using the multi-primer PCRs (consensus primer cycle threshold (Ct) values *versus* (*vs*.) multi primer Ct values 9.2 *vs*. 8.7 for *SjR2* and 14.0 *vs*. 7.4 for *SjCHGCS19*). Adding this additional sensitivity increase to the limits of detection as obtained with the positive control plasmids (see methods), copy numbers of the targeted retrotransposons as they can be expected within a single helminth cell were within the sensitivity range. The multi-primer PCRs were, accordingly, included in the application with residual serum samples from patients with *S. mekongi* infections. The oligonucleotide sequences of the various PCR primers and probes are shown in Table 1.

### 3.2. PCR Reactions with Residual Serum Samples from Patients with S. mekongi Infections

Positive or borderline positive real-time PCR signals were achieved in 6 out of 61 (9.8%) residual serum samples from patients with *S. mekongi* infections, as collected in the course of recent studies [32,33,34] using multi-primer PCRs, while no signals were obtained with consensus primer PCRs. In detail, an unambiguously positive result (serum Ct 35) was obtained with the multi-primer *SjR2* real-time PCR in a patient with 228 *S. mekongi* eggs per gram stool, another borderline result (serum Ct 38) in a patient with 60 eggs/gram. With the *SjCHGCS19* multi-primer real-time PCR, 4 borderline positive signals with Ct values of 37, 38, 44 and 45, respectively, were recorded in serum samples from patients with 1110, 24, 36, and 132 *S. mekongi* eggs per gram stool, respectively. There was no matching regarding the positive signals obtained with both multi-copy PCR assays. Patients with positive or borderline positive signals in *S. japonicum* complex-specific real-time PCR showed egg counts per gram stool higher than the median value of 18 eggs/gram in all instances and higher than the mean value of 51 eggs/gram in 4 out of 6 instances, respectively.

### 3.3. PCR Reactions with Negative Control Residual Serum Samples from Patients with S. mansoni or S. haematobium Infections

In 50 residual sera from patients with *S. haematobium* or *S. mansoni* infection [12], the *SjR2* multi-primer real-time PCR did not show any cross-reaction. The *SjCHGCS19* multi-primer real-time PCR showed two positive results (Ct values 31 and 35, respectively) in the serum of two patients with double infections due to *S. haematobium* (serum Ct values 32 and 20 in *S. haematobium*-specific *Dra1* real-time PCR, respectively) and *S. mansoni* (serum Ct values 39 and 23 in *S. mansoni*-specific *Sm1-7* real-time PCR, respectively), as well as one borderline positive result (serum Ct value 36) in a patient with *S. mansoni* (serum Ct value 33 in *S. mansoni*-specific *Sm1-7*-PCR). As shown for these three samples, differences between the Ct values of *S. mansoni* complex-specific *Sm1-7* real-time PCR, *S. haematobium* complex-specific *Dra1* real-time PCR and *S. japonicum* complex-specific multi-primer *SjCHGCS19* real-time PCR were highly variable ranging from +1 to -12. Additionally, there was no obvious hint for a preferential cross-reaction of *SjCHGCS19* real-time PCR in samples with particularly low *Sm1-7* and *Dra1* Ct values, as cross-reactions were also recorded in samples close to the Ct mean values of the negative control sample collection as observed with *S. haematobium* complex-specific and *S. mansoni* complex-specific PCR.

In the two assessed urine samples positive for *S. haematobium* in *Dra1* PCR, the sample with the *Dra1* Ct value of 25 and microscopically visible *S. haematobium* eggs showed a positive *SjCHGCS19* multi-primer real-time PCR result (Ct value 34), while the other urine sample with a *Dra1* Ct value of 30 went undetected in the *SjCHGCS19* assay.

## 4. Discussion

Hybridization-probe-based real-time PCR targeting multi-copy sequences is a well-established approach for the diagnosis of infections with parasites of the *S. haematobium* complex and the *S. mansoni* complex in human serum [2,7,8,9,10,11,12,13,14]. To facilitate the diagnosis of schistosomiasis caused by parasites of the Asian *S. japonicum* complex, calls for action to develop similar diagnostic options were published earlier [34]. The retrotransposons *SjR2* and *SjCHGCS19* were identified as potential targets [17,19] due to their high numbers of repeats within the genomes of members of the *S. japonicum* complex [19]. In line with this, these sequences have been repeatedly used for the diagnostic detection of *S. japonicum* [19,20,21,22,23,24,25,26,27,28,29,30]. However, hybridization-probe-based real-time PCR assays with human serum targeting not only *S. japonicum* but also *S. mekongi* and *S. malayensis* have not yet been broadly evaluated so far.

In the study presented here, we provided the proof of principle that *SjR2*- and *SjCHGSC19*-based real-time PCR can be used to diagnose *S. mekongi* infections in human serum. Sensitivity was, however, poor with positive or borderline positive PCR signals in less than 10% of the assessed historical serum samples from patients with microscopically proven *S. mekongi* infections. However, even this low sensitivity was only reached by combinations of primers targeting different variants of the retrotransposons. Further, parallel microscopic stool assessments from the study participants suggested that positive serum PCR signals were predominantly observed in patients with more severe infections, as demonstrated by higher loads of *S. mekongi* eggs per stool volume. Even in those more severely infected individuals, however, the positive results of *SjR2*- and *SjCHGCS19*-specific PCRs did not match directly, suggesting target DNA concentrations close to the limit of detection. More than this, even the sensitivity of the multi-primer PCR assays as described here could be achieved by several adaptations of the oligonucleotides only.

While *SjR2*-specific PCR did not show any cross-reaction with serum and urine samples positive for the African *Schistosoma* species *S. haematobium* and *S. mansoni*, such cross-reaction was inconsistently observed for the *SjCHGCS19*-specific multi-primer PCR. Considering the low sensitivity of *SjR2*-PCR even in serum of *S. mekongi* patients, however, this seemingly better specificity is difficult to interpret. Nevertheless, the recorded cross-reactions of the *SjCHGCS19*-PCR are not surprising as well, as positive results of *SjCHGCS19*-specific PCRs in samples from patients with *S. mansoni* infections were already reported by Guo and colleagues [19]. Insofar, the results of the specificity testing with serum samples from patients with infections due to phylogenetically related *Schistosoma* spp. such as *S. mansoni* had been expected. Here, we were able to show that *S. haematobium* is detected by *SjCHGCS19*-PCR as well. Interestingly, only a few samples showed such cross-reactivity, and there was no obvious association with parasite DNA loads, as suggested by *Sm1-7*-PCR and *Dra1*-PCR with specificity for *S. mansoni* complex and *S. haematobium* complex, respectively. In contrast, the observed differences between Ct values of *SjCHGCS19*-PCR and *Sm1-7*-PCR or *Dra1*-PCR were highly variable among the few samples with overlapping positive results. This observation suggests high sequence variability of the *SjCHGCS19* sequences within non-*S. japonicum* complex schistosomes, explaining the apparent inconsistency of positive *SjCHGCS19*-PCR results.

The reasons for the much lower diagnostic sensitivity of *SjR2*-PCR and *SjCHGCS19*-PCR for the detection of *S. japonicum* complex PCR in human serum compared to PCRs for other highly repetitive multi-copy targets like *S. mansoni* complex-specific *Sm1-7*-PCR or *S. haematobium* complex-specific *Dra1*-PCR [2,7,8,9,10,11,12,13,14] remain speculative. High genetic intra-species variability of the retrotransposons and varying numbers of repeats within individual worms as described elsewhere [18,19] might make *SjR2* and *SjCHGCS19* sequences less suitable as real-time PCR targets, at least for the diagnosis from human serum.

For the diagnosis from other sample materials such as stool, however, less complex target sequences like ribosomal gene sequences of *S. japonicum*, for which hybridization probe-based real-time PCR has already been introduced [40], may be sufficient. The multi-primer PCRs for *SjR2* and *SjCHGCS19*, in contrast, should not be applied in complex samples like stool specimens from patients with a high likelihood of various helminth infections prior to thorough evaluations of these assays with stool samples obtained from such settings. The latter would have been beyond the scope of the present work, and we also did not have suitable samples for this; however, specificity issues of multi-primer PCRs applied with stool samples are known from other previous assessments performed with group-specific helminth PCRs [41].

In human blood, in contrast, highly repetitive multi-copy targets seem to show superior sensitivity, as recently demonstrated in a comparison of ITS-2-based real-time PCR and *Sm1-7*-based real-time PCR for the diagnosis of *S. mansoni*-specific DNA in human EDTA blood samples [12]. Another hypothetical reason for the poor scoring of the *S. japonicum* complex-specific multi-primer PCRs might be reduced circulation of free helminth DNA in the peripheral blood in case of *S. japonicum* complex infections compared to infections with *S. mansoni* or *S. haematobium*. The design of this study, unfortunately, did not allow to answer this question.

It remains speculative whether or not DNA extraction from high serum volumes up to 20 mL as earlier suggested for *S. mansoni*-specific *Sm1-7*-serum-PCR [7] might have increased the diagnostic sensitivity of *SjR2*-PCR and *SjCHGCS19*-PCR. Respective high residual sample volumes, however, were not available for the evaluations presented here.

Altogether, the limited availability of serum samples from patients with confirmed *S. japonicum* complex infections both in numbers and regarding their residual volumes was the main limitation of the study. This restriction also prevented the assessment of different nucleic acid extraction strategies, which may hypothetically have led to improved sensitivity. Additionally, the low volumes made repetitive testing impossible because the eluates were rapidly gone after just a few PCR runs. Another limitation was the need for relying on historical serum reference sample materials due to the scarce availability of fresh samples from patients with *S. japonicum* complex infections. Although real-time PCR targeting the human 18S rRNA gene [35,36], which was performed from randomly chosen samples to estimate the DNA quality (see methods), did not suggest considerable degradation of human DNA within the samples, it can only be speculated whether this also applied to free helminth DNA. As the *S. mekongi* serum samples had originally been collected for serological test purposes and not for DNA detection, they had been treated accordingly in the diagnostic process and stored at −80 °C with several freeze-thaw cycles. Further, it remains uncertain if the late stage of infection, as indicated by the shed eggs of the patients, might also have contributed to the observed low sensitivity. As known from the *Sm1-7*-specific real-time PCR, the amount of *Schistosoma* spp.-DNA in serum varies depending on the stage of infection [34]. Finally, due to the lack of suitable specimens, only serum samples from patients with *S. mekongi* were assessed. Accordingly, the evaluation presented here does not comprise all species of the *S. japonicum* complex. However, due to the high genetic similarity of the target sequences in the different species as suggested by the obtained next generation sequencing results (see Methods and Appendix A), it is likely that the PCR results will be similar using serum samples from patients infected with *S. japonicum* or *S. malayensis*.

Although mainly negative results are presented, the experiences described in this study may guide the future development of *S. japonicum* complex-specific PCRs from human serum.

## 5. Conclusions

The assessment allowed the proof of principle for the use of hybridization probe-based real-time PCR for the detection of *S. japonicum* complex in human serum samples by targeting retrotransposon sequences. Low positivity rates in residual materials of clinical samples, however, leave room for further improvement and calls for evaluations with fresh sample materials. The experience from this work may help to guide future test development and evaluation approaches in areas of endemicity.

## Figures and Tables

**Table 1:** Oligonucleotides applied in the consensus PCRs and multi-primer PCRs targeting the *SjR2* sequence and the *SjCHGCS19* sequence of *S. japonicum* complex DNA.

Forward Primer Name	Forward Primer Sequence	Reverse Primer Name	Reverse Primer Sequence	Probe Name	Probe Sequence
**Consensus Primer PCR Targeting** **the *SjR2* Sequence (Primer Sequences Adapted from [17]** **)**					
SjR2 F	5’-GAGGAAACCGAAAGGCACCTA-3’	SjR2 Ri	5’-GCTAATAGCAATGTGGTCTATTTGAGTCCA-3’	SjR2 Asia P	5’-CAGTAGGTAACTGTTTAGACTTATCAAGGAAACG-3’
**Multi-Primer PCR Targeting** **the *SjR2* Sequence**					
SjR2 Smek F1 old	5’-CGAGAGCGTGGGCGTTAGA-3’	SjR2jap1(16) R	5’-GTCCAGCGTTGGGTTGATTTT-3’	SjR2 Asia P	5’-CAGTAGGTAACTGTTTAGACTTATCAAGGAAACG-3’
SjR2 F1	5’-GGTGATTTTAATGCCCAAGTAGGTAGA-3’	SjR2jap4(45) R	5’-GTCCAGCGTTGAGTTGATTGT-3’		
SjR2 Smek F1	5’-GGTGATTTTAATGCTCAAGTAGGTAGA-3’	SjR2jap5(262) R	5’-gTCCAACgTTgAgACgATTTT-3’		
SjjR2jap10 F1	5’-GGTGACCTTAATGCTCAAGTAGGTAAA-3’	SjR2jap7(11) R	5’-GTTCAACGTTGAGACGATTTT-3’		
SjR2 F	5’-GAGGAAACCGAAAGGCACCTA-3’	SjR2 Ri	5’-GCTAATAGCAATGTGGTCTATTTGAGTCCA-3’		
SjR2 Smek F	5’-GGCGAAACTGAAAGGCACCTA-3’	SjR2 Smekold Rii	5’-AATTCTAGCAATGTGGTCTATTTGAGTCCA-3’		
SjR2jap F lg	5’-GGTAGACTAAGTCAGACTGAAAGACACTTA-3’	SjR2 Smek Rii	5’-TGATAGCAATGTGGTCTATTTGAGTCCA-3’		
SjR2jap4 F (300)	5’-AGTGAAAATGAGAGACACTTG-3’	SjR2jap1(10) Rii	5’-GGCGATGTGATCTAGTTGGGTTCA-3’		
SjR2jap5 F (136)	5’-GACCAAACAGAAAGACATTTA-3’	SjR2jap3(252) Ri	5’-GGCGATGTGATCTAGTTGGGTCCA-3’		
**Consensus Primer PCR Targeting** **the *SjCHGCS19* Sequence (Primer Sequences Unchanged from [19]** **)**					
Guo-SjCHGCS19 F	5’-CCAAATCGCAACACTACG-3’	Guo-SjCHGCS19 R	5’-ATCGGATTCTCCTTGTTCAT-3’	Guo2012 P	5’-AATGGAACTCGTCAYTGTACATCAACTTCA-3’
**Multi-Primer PCR Targeting** **the *SjCHGCS19* Sequence**					
7436 F	5’-ACCCACCAGWCATCGAAGCA-3’	9292 A	5’-RGCCTGTTGATCTCGAAGTTG-3’	9015 P	5’-ACCAGGRAAAGTCTTCAACAGAGTGTT-3’
7443 F	5’-CCACCAGWCATCGAAGCAGC-3’	9292 R	5’-RGCCTGTTGATCTCGAAGTT-3’	3483 P	5’-CACCAACGATGRAAGAAATCAAGATRGYY-3’
2980 F	5’-RARAAAGCTGCAAGAGAAGG-3’	3604 R	5’-TCCTGCTGCTTTCCCACTTT-3’		
2983 F	5’-RAAAGCTGCAAGAGAAGGAA-3’	3613 R	5’-TGTCAGGTCCTGCTGCTTTC-3’		
3956 F	5’-ACAGAGGRATCACACTRTTGTCAGT-3’	3615 R	5’-TTGTCAGGTCCTGCTGCTTT-3’		
3956Sma F	5’-ATAGGGGGATCACACTACTGTCAGT-3’	4113 R	5’-RAAGTTGAGCATCCACTGAR-3’		
3956Sme3 F	5’-AAAGAGAGATCACGCTACTGTCGAT-3’	4113Sma R	5’-AAAGTTAAGTACCACTGAA-3’		
3956Sme3 F I	5’-ACATAGAGATCACACTACTGTCGGT-3’				

Design-related numbers are indicated by brackets, references by square brackets.

## Data Availability

All relevant data are provided in the manuscript. Raw data can be made available on reasonable demand.

## Appendix A

**Table A1:** ***SjR2*-like sequences and*****SjCHGCS19*-like sequences identified in***S. mekongi* and *S. malayensis* cells available in reference material at the Bernhard Nocht Institute for Tropical Medicine, Hamburg, Germany.

**Contig of a *Sjr2*-Like Sequence as Identified in both *S. mekongi* and *S. malayensis***
5‘-CCCTAGATTCTCGCTCGATCGATGTCTGCTGCGTCTCCGAAATGCATATACAGGATCCAAGTACAAACATTCGCTCGACCTCACCTTGTAAAGATAAAGAAGCGGTCCGGTTCACCCTTCGTGTTTCCGGAGACGCCATCGCTACCACTCGAGGCCTTGCTAGTGTAGGCATAGCACTAAATTCAAAAGCTGAACAGGCTCTCCTTCACTGGATTCCCATAGATAGTCGCTTATGCGCTGTCCGTCTAAACGGGAGGGTAAGAACTCGTAAAGATAGGGACACACGCCGTTGCGTTTTAGTTATGTCTGCCTAAGCTCCCAGTGATTGAAGCTCTGATGAAGTAAAAGATTAATTTTACAGAAAGCTTTCCGACCTCCTTCGGAAAGCTAAGAGTACTGATGTAGTGATCGTGGCTGGTGATTTTAATGCTCAAGTAGGTAGATTAGGCGAAACTGAAAGGCACCTAGGTGGATCCTATGGTGTCGAGGCTCAACGAACAGACAATGGCGACCGTCTGTTACAACTATGTTCGAATAACCATCTATTTCTCGCAAGCACCAACTTTAAATATAAGGAAAGACATCAGTTGACACAGCGGCCCCCACAGTCAGCTCAACGTTGGACTCAAATAGACCACATTGCTATCAGCCACCGTTGGAGAGGCTCGATAGAAGACTGCCGCTCATTTTGGAGCACATGTTTAGATTCAGACCATGCTCTAGTACGAGCGCGTATTAGTCTGCGTCTCACTGGACGTAAAGGATCCACGGCAAGGATCCCCGTCAGAATTCAACTTAATGATGAAAAAGTCAAAAATACATTTTAGGAACAACTAGAAAACCAATTGGTCAACTGTGTAAGCCATACCCATCCCGAGTCAGCTTGAAATGATATCCAAAAAGCTGTTAAAACAGTAGTAATGTCTACTAGTAAACCAAGCCATAAAGCTAAGAAAACTCAGTGGATTTCAGCTGCGTCTACTGAACTGATAGATGCTCAGCAACACATCCCGTCTAGTTCTAAATAAGATGAGGAGCGAAGGCAGCCTAAACGCAAACTGGTTAAAAGCTTACGCAATGATCGTGAGCAGTGGTGGGTGGCGAAAGCTGGAGATGGAAAAGACAGTGGCAATAGGTAACAGTAGGCAACTGTTTAGACTTATCAAGCAAACGGGTATCAGAAATCACATTGTAAGTAAAATTATCTCAGAAAAGGATGGGAGCATTATTCACTGTCAATCCAGAAGCTTGGACCGATGAGCAAAACACTTTAGGGAACAGTTCAAGTGACCCTCGGCCTCATACCAGTTGCCCACCATTCCCAAGCAATCGGAATGGCAAGTTAATATTGGTCCCCCAAGTCTTAGTGAGGTTGAGAAGACTATAGGAAATCAAAAGCGAGGGAGAGCAGCAAGACCTGATGGATTAACCCTTGAGATATTTAAGGATGGTAGT-3‘
**Contig of a** ***SjCHGCS19*-Like Sequence as Identified in *S. mekongi* Variant 1**
5’-GGGCTAGCACTCCCATCCCTGAAAACACAAGTTTGCTAAAAAAAAAACGATAACCAGATTAAACAAGCTAAACCATTTAAACTCTGTCCTGAGAGTGGAAGGAATGATTATGACGCCTCATGGTGAAATCCGAGATTCTTTGGAATTCATGAGGCCGATGCCCCTTCTAACAACCAGAGTAAAACTTTTTATAGGTGAGTGGGATATTCGAACAATGTGGAAGAGCGGGAAGACCAATCAAATAGCAGCAGATATAAAGAGATACAACTTGACAGTGCTCGGAATCAGTGAAACCCAGTGGACTCAAGTTGGATAGAAAACATTGGCTACGGGAGAGATGCTACTATACTCCGGTCACGAAGAGGAAAATGCTTCTCACACCCAAGGACTTGATCTGATGCTGTCCAAAACAGCACGTAATGCACTTGTGGGATGGGAACCACGTGGACCCAGGATCATCAAAGCATCTTTCAAAACAATGAAGAAGGGGATTACAATCAATATTATCCAATGTTATGTACCCACCAATGACGGCAACGACGAGGACAAAGATCAGTTTTATGATAGGTTGCAATCAATTATGGCGAAGTGTTCAGGAAAGGACCTGACAATCCTGATGGGGGACCTAAACGCCAAAGTTAAAATGGACAACACCAGATATGAAGAAATCATGGGACGACAGGGACTGGGAGAAAGAAATGACAATCTTGAAAGATTTACAAACCTATGTGCTTTCAGTAAAATGGTTAAAGGTAATACAATATTCCCCCCCCCAAACAAACCCATACACAAGGCTACATGGGTCTCATCAGATCATACTAAAGAGAATCAAATCGATCATATATGCATCAATAAAAAATTCAGATCAATCGAAGATGTGCGAACAAGGAGAGGAGCTGACATAGCTTCAGATCACCACCTGGTGGTTACTAAAATGAAACTGAAGCTAAAGAAGCAGTGGACAACTGGAGTAACAGCAGTACGACGGTTCAATAAAGCCTTTATTCGAGATACTGACAAACGCAAGGAGTTCGAAATAGCTCTCAACAACAGGTTCCAAGCTTTACAAGATCTACTGAAAAAAGAAACTACTATGGGGCGAAACTGGAAAGGAATCACAGAAGCACTAACTTCAACGTGTCAGGGGGTTCTGGGCCGAAAACAGCACCATTATAAGTAGTGAATTTTCACTGAAACATTGAGGAAAATTCAGCAAAGGAGGAACAAGAAGACATAAATCAACAACAGCGGAAC-3’
**Contig of a** ***SjCHGCS19*-Like Sequence as Identified in *S. mekongi* Variant 2**
5’-AACAACTGGTTCCAAGCCTTACAAGGTCTACCGAAAGAAGAAGAAACTACCATTCGGGACAACTGAAAAGAAATCATAGAAGCACTAACGTCAACGTGTCAGGAGGTTCTGGGCCGCAAAAAGCATCATTAAAAGGAATGGATCTCCAATGAGACATTGAGCAAAATTCAACAAAGAAGGAACAAAAAGACAGAAATCAACAATAGCCGAACAAGAGCAGAGAAAATCAAGGCACAAACTGAATACACAGAAGCAAACAAACAAGAAGAGCACTATAGCCGACAAACACAAATACATTCGAAGGCCTTGCAACGACAGCGGAAAAAAGCTGCAAGAGAAGGAAATATGAAACAACTATGTGACACAACGAAACATCTGGCAGGCAAATATAGTAAACCAGAGCGACCGATCAAGAACGAAGAAGATAAGCCAATCACCGAGAATTAAAAGCAAAGAAACAGATGGGTAGAATACTTTGAGGGACTCCTAAGTAGGCCAGCTCCACTGAGCCCACCAGGCATCGACGCAGCACCTGCAGATCTCCCGATAGATATCACTCCACCAACAATAGAAGAAATCAAGATGGCCATCAGACAAATCAAAAGTGGGAAAGCAGCAGGATCTGACAATATGCCAGCTGAAACATTCAAGTCAGATATAGATGTAACGGCAAGGATACTCCACGTTTAATTTAGGAGGATCTGGGAGGAAGAACAAGTGCCAGCAGACTGGAAAGAAGGACACCTTATCAAGATGCCAAAGAAAGGAGATTTGAGCGAATGTGAGAACTACATAGAGATCACACTACTGTCGGTACCAGGAAAAGTCTTCAACAGAGTGTTGTTGAACAGAATGAAAAATTCAGTGGATGCTCAACTTCGAGATCAACAGGCTGGTTTCCGTAAGGATTGGTATTTCACAGACCAAATCGCAACACTATGGATTATCGTTGAACATTCAATTGAGTGGAAATCGTCACTGTACATCAACTTCACTGATTATGAGAAGGCGTTTGACAGTGTAGATAGGAGGACACTATGGAAACTTCTTCGACACTACGGCGTACCAGAGAAACTCGTCAACATCATCCGGAACTCTCACGACGGACTACAGTGCAAAATCGTGCATGGAGGATAGCTGAAAAATGCATTCCCAATGAAGACCGGAGTCAGACAAGGTTGTCTACTCTCGCCATTCCTCTTTCTTCTAGTAGTTAACTGGATCATGGAGACCTTCACATCTGAG-3’
**Contig of a** ***SjCHGCS19*-Like Sequence as Identified in *S. mekongi* Variant 3**
5’-GCAAGGATACTCTACGTTTTATTCAAAAAGATCTGGAAGGAAGAACAAGTGCCGGCAGACTGGAAAGAAGGACATCTTATCAAGATACCTAAGAAAGGAGATTTGAGCAAATATGAGAACTAAAGAGAGATCACGCTACTGTCGATAGCAGGGAAAATCTTCAACAGAGTATTGTTGAACAGAATGAAAGATTCAGTGGAGGCTCAACTTCGGGATCAACAGGCCGGTTTTCGTAAGGATCAGTCGTGCACAGACCAAATCGCAACACTACGGATCATCGTTGAACAATCAATTGAATAGAACTCGCCACTGTACATCAACTTCATTGATTATGAAAAGGCGTTTGATAGTGTAAATAGGAGAACACTATGGAAACTTCTTCGACTCTACGGCGTACCAGAAAAACTCGTCAACATCATCCGGAACTCTTACGACGGAACACAGTGTAAAATTGTGCATGAAGGACAGCAGACAAATGCATTCACAGTGAGGACCGGAGTCATACAAGGCTGCCTACTCTCGCCTGTTCTCTTCCTTATAGTGGTTGACTAGATTATGAAGACCTCCACATCTGAGGGGATGCACGGAATTGAATGGACAAGTAGGATGCAACTCGATGATTTGGACTTTGTGGATGATCTAACTTTCTTAGCACATACACATCAACAAATGCAAGCAAAGACAACCAGTGTAACAGCAACTTCAGCATCAGTAGGCCTGAACATACATAAGGGAAAGAGCAAGATTCTTAAATACAACATAGGGAGCACCAACCCAATTCAACTTGATGGAGAAACTCTGGGAGAAGTGGATACTTTTATATACTTGGGGAGCGTTATCGATGAACAAGGAGGGTCGGAGGAAGATGTAAACCCAAAGATTGGTAAAGCCAAGGCAGCATTTCTACAGTGAAAAACATATGGGACTCAAAACAACTGTCAACTAACCTCAAAGTCAGACTCTTTAATTCGAACGTCAAAACAGTTCTGCTATATGGAGCTGAAACATGGAAAATCACTGCAAACATCCTCAGAAGGGTACGAGTATTTATAAACAACTGCCTACACATGATATTGACTGTCCGTTGGCCGAAAACCATCAGCAACGGCTTACTATGGGAGAGGACAAACCAACTTCCTTTCGAAGAGGAAACTAGGATAAGACGTTGGGAATGGATAGGACAATCACTGCTGAAATCATGAGACTGCATCACTAGACAAACATTAACATGGAATTTTGTTGGAAAACGTAAAAGAGGAAGGCCAGAGAACACCCGGTGTGGAGATTGGAAACAGAAATTTAAAAAGAAAAAAGGAACCGGAAAAACCTGGAACGGATGGCCCAGGGAGAGTTTAA-3’
**Contig of a** ***SjCHGCS19*-Like Sequence as Identified in *S. malayensis***
CGGGAGAGATGCTATTATACTCCGGCCACGAGAGGGAAAATGCTTCTAACACCCAAGGAGTTGATTAAATGCTGTCCAAAGAAGCACGTAATGCATTTGTGGGATTAGAACCACATGGACCCAGGATCATCAATGCATCTTTCAAAACAATGATGAAGAGGATTACAATGAATACTATCCAATATTATGCACTCTCCAACGATAGCAACGATGAGGATAAAGATCGATTTTATGGTAGGCTATAGTGAAGTAGTTATAGTGAAGTGCCCAGGAAAGGATCTGATAATTCTGTTGGGGGGGTTCCTAAACCCCAAAGTCAGAATGAACAACTCCGGATATGAAGAAATCATGGGACGACAGGGACAGGGAAGAAATGAGAATGGTGAATTATTTACAAATCTATGTGCTTTCAACAAAATGGTTATAGGTGGTACGATATTCTCCCACAAACGCATACACAAGGTTACATGGAGCTCTTCGGATCATACTACAGTGAATCAAATCGATCATATATGTATCAATAAAAAATTCAGAAGATCAATGGAAGGTGTGCGAACAAGGTGAGGAGTTGATATAGCATCAGACCACCACCTGGTGGTTGCTAAAGTGAAACTGAAGCTAAAGAAGCAATGGACAACTGGAGTGTCAGCAGTACAACGGTTCAATACAGCCTTTATTCAAAATACTGGCAAACTTAAGGAATTAAAAATAGCTCTCAACAACAGGTTCCAAGCCTCACAAGATCTACTGAAAGAAGAAGAAACCACTATGGAGGACAACTGGGGAGGAATCAGAGAAGCACTAACTTCAACGTTTCAGGGGGGTTCTGGGCCGCAAAAAGCACCATTATAAGGAATGGATCTCCACTGGGACCCTAAGTAAAATTCAATAAAGGAGGAACAAGAAGACAGAAATCAAGAACAGCCGAACAAGAGCAGAGAAAATCATTGTACAAACTAGATACACAGAAGCAGATAAGCAAGTAAAGAAGAGCATTAAATCCGACAAACAGAAACACATCGAAGGCCTTGCAACGATAGCAGAGAAGGCTGCAAGAGAAGGAAATATGAAACAGCTATACGACACAACGAAGAAACTGGCAGATAAACATGGTAAACCAGAGCGACCGGTCAAGAACAAAGAAGGCAAGACACTCCCTGAAATTCAAGAACAAAAGAATAGGTGGGTAGAACACTTTGAGGAACTCTTGAATAGGCCAGCTCCACTGAACCCACCGGACATCAAAGCAGCACCTACAGATCTCCCGATAGATGTCACTCCACCAACGGTGGAAGAAATCAAGATGGCCATCAGACAAATCAAAAGTGGGAAAGCAGCAGGACCAGACGATATACCAGCTGAAGTACTCAGGTCAAACACAGATGTAACAGTAAGGATGCTCCATGTTTTATTTAAAAAGATCTGGGAGGAAGAACAAGTGCCGACAGATTGGAAGGAAGGACATCTTATAAGAATTACAAATAAAGAAGATTTAAGAAAATGTGAGAACTATAGGGGGATCACACTACTGTCAGTACCAGGGAGTCTTCAACAGAGTGTTGCTGAACAGAATGAAAGATTCAGTGGTACTTAACTTTGAAATCAACAGGCTGGTTTTCGTTAGGATCGTTCGTGCACAGACCAAATCGCAACACTACGGATCACCGTTGAACAATCAATTGGATGGAACTCGTCACTGTACATCAACTGCATTGGCTATGAGGTATTTGACAGTGTGGACAGGAGAACACTATGAAAACTTCATCGACACTACATCGTACCAGAGGAACTCGTCAACATCATCTGAAACTCTTACGACGAAACACAGTTCAAAGTTGTTCATGGGGGGACAGCTGACAAATACATTCCCAATGAAGACTGTAGTCAGACAAGGCTGCATACTCTCGCCTTTTCTCTTCCTTCTAATGGTTGATTGGATCATGAAGACCTCCACATCTGAGAAAATGCACAGAATTCAATGAACAAGTAGGATACAATTCGATGAATTGGACTTCGCGGATGATCTAGCTCTCCTAACACATATACACCAACAAACACAAGTGACGACAACCAACGTAGCAGCAACCTCAGCATCAGTAGGCCTGAACATACATAAGGGAAAGAGCAAGATCCTCAAATACAACACAAGGAGCACTAACCCAATTGAATTTGATGGAGCAACTCTGAAAGAAGTGGATGCTTTTACGTACGTGGGGAGCATCATCGATAAACAAAGAGGGTCCGACACAGATGTAAAGGCAAGGAATGGCAAAGCCAGAGCAGCATTCCTACCTTTGAAAAACATGTAGGATTTAAAACAAATGTCAACGAACTTCATAGTCAGACTCTTCAATTTGAACATGTAAACAGTTCTGTTGTATGGAGCTGAAACGTGGAGAACTACTGTAAACATCATTAGAAGAGTACATGTATTTTTAATCTACTGTCTACACATGATACTGAACGTCGGTTGGCCGGGAACCATCAGCAACAGTTTACTATGGGAGAAAACAAACCAACTTCCAGCTGAAGAGGAAATTAAGAAAAGACGTTGGAAATGGATAGGACGTTCATTACCATCAGACTGCATCACGAGACAAGCATTAACTTGGAATCCTGTTGGAAAACGGAAAAGAGGAAGGCCAAAGAACACACTGCATCTAGAGTTAGAAGCAGATATTAGAAGGATGAACAGCAACTGGAAACAATTGGAAAGGATTGCTCAGGACAGAGTCCGATGGAGAGTACTGGTGAGGGGGCTATGCTCCTCTGTGAGCGGTAACGGGCTTAAGTAAGT
